# Non-cysteine linked MUC1 cytoplasmic dimers are required for Src recruitment and ICAM-1 binding induced cell invasion

**DOI:** 10.1186/1476-4598-10-93

**Published:** 2011-07-28

**Authors:** Ashlyn J Bernier, Jing Zhang, Erik Lillehoj, Andrew RE Shaw, Nirosha Gunasekara, Judith C Hugh

**Affiliations:** 1 Department of Laboratory Medicine and Pathology, 3-70 Heritage Medical Research Centre, University of Alberta, Edmonton, AB, T6G 2S2, Canada; 2 Department of Pediatrics, University of Maryland, School of Medicine, Baltimore, MD, 21201, USA; 3 Department of Oncology, Cross Cancer Institute, University of Alberta, Edmonton, AB, T6G 1Z2, Canada

## Abstract

**Background:**

The mucin MUC1, a type I transmembrane glycoprotein, is overexpressed in breast cancer and has been correlated with increased metastasis. We were the first to report binding between MUC1 and Intercellular adhesion molecule-1 (ICAM-1), which is expressed on stromal and endothelial cells throughout the migratory tract of a metastasizing breast cancer cell. Subsequently, we found that MUC1/ICAM-1 binding results in pro-migratory calcium oscillations, cytoskeletal reorganization, and simulated transendothelial migration. These events were found to involve Src kinase, a non-receptor tyrosine kinase also implicated in breast cancer initiation and progression. Here, we further investigated the mechanism of MUC1/ICAM-1 signalling, focusing on the role of MUC1 dimerization in Src recruitment and pro-metastatic signalling.

**Methods:**

To assay MUC1 dimerization, we used a chemical crosslinker which allowed for the detection of dimers on SDS-PAGE. We then generated MUC1 constructs containing an engineered domain which allowed for manipulation of dimerization status through the addition of ligands to the engineered domain. Following manipulation of dimerization, we immunoprecipitated MUC1 to investigate recruitment of Src, or assayed for our previously observed ICAM-1 binding induced events. To investigate the nature of MUC1 dimers, we used both non-reducing SDS-PAGE and generated a mutant construct lacking cysteine residues.

**Results:**

We first demonstrate that the previously observed MUC1/ICAM-1signalling events are dependent on the activity of Src kinase. We then report that MUC1 forms constitutive cytoplasmic domain dimers which are necessary for Src recruitment, ICAM-1 induced calcium oscillations and simulated transendothelial migration. The dimers are not covalently linked constitutively or following ICAM-1 binding. In contrast to previously published reports, we found that membrane proximal cysteine residues were not involved in dimerization or ICAM-1 induced signalling.

**Conclusions:**

Our data implicates non-cysteine linked MUC1 dimerization in cell signalling pathways required for cancer cell migration.

## Background

The ability of malignant cells to escape from a primary tumour mass and migrate to distal sites to form metastatic tumors is the cause of mortality in the majority of carcinomas, including breast carcinoma. Approximately 20% of breast cancers belong to the Luminal B genetic subtype, typified by estrogen receptor positivity and a slow, steady rate of recurrence over time despite anti-estrogen therapy [1]. Estrogen is known to increase the expression of MUC1 [2], a well-characterized member of the mucin family of glycoproteins, and a correlation has been demonstrated between MUC1 expression, resistance to anti-estrogen therapy and metastatic behaviour [3]. We have been investigating the mechanism of cell migration in the Luminal B breast cancer cell lines MCF7 and T47D, and were the first to demonstrate that MUC1 mediates heterotypic cell-cell adhesion by binding ICAM-1 [4], which is expressed on peritumoral stromal and endothelial cells. Subsequently, we demonstrated that ICAM-1 binding triggers calcium oscillations which may activate proteins involved in focal adhesion disassembly and cell contraction. In keeping with this, we further reported that after interaction with ICAM-1, transendothelial migration invasion in MUC1 expressing cells is associated with increased MUC1-Src association, MUC1-cytoplasmic domain (MUC1-CD) phosphorylation, CrkL recruitment, and Rho-GTPase mediated cytoskeletal rearrangement [5-7].

**Figure 1:**
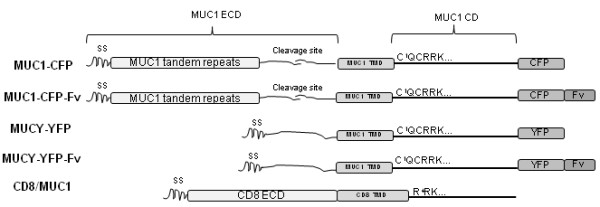
**Schematic of constructs used in this study**. "SS" indicates signal sequence, "ECD" indicates extracellular domain, "TMD" indicates transmembrane domain and "CD" indicates cytoplasmic domain. On SDS-PAGE, full-length MUC1 dissociates at "cleavage site" and runs as two separate entities.

MUC1 (also known as DF3, CA15-3, or episialin) is expressed apically on normal breast epithelia, but often loses this polarization and becomes underglycosylated in breast cancer [8,9]. MUC1 is translated as a single polypeptide, followed by conformational stress-induced cleavage resulting in a heterodimer of non-covalently associated extracellular and cytoplasmic portions [10,11] (Figure 1). The extracellular portion consists of a variable number of 20-amino acid (aa) tandem repeats containing multiple sites for O-glycosylation, which impart a negative charge and result in a structure that can extend up to 500 nm from the cell surface. The cytoplasmic portion consists of a 58-aa extracellular stub, a 28-aa transmembrane domain, and a 72-aa cytoplasmic domain, which contains seven conserved tyrosine residues, and has been shown to interact with diverse effectors [Reviewed in [12]] which is important since MUC1-CD itself lacks tyrosine kinase activity.

The signalling capacity of transmembrane proteins lacking kinase activity is often mediated by associated non-receptor tyrosine kinases. In some instances, these kinases are bound to pre-formed dimers of the receptor [[13], Reviewed in [14]]. Upon ligand binding, structural changes such as cysteine linkage, association with detergent resistant membrane fractions, and changes in cleavage result in signal initiation [15-17]. Previous work by others has demonstrated that constructs of the MUC1-CD form oligomers *in vitro *which are disulfide-linked, and *in vivo *which are dependent on the membrane-proximal cytoplasmic C^1^QC motif [18,19] (Figure 1). Here, we investigated dimer formation in wild-type MUC1 and the relationship between dimerization, Src recruitment and ICAM-1 induced signalling events. We also examined the role of membrane-proximal cytoplasmic domain cysteine residues in these phenomena. We confirm that Src is an essential mediator of the previously observed ICAM-1 binding pro-motility events and show that MUC1 forms constitutive cytoplasmic domain dimers which are required for constitutive Src recruitment and ICAM-1 binding induced signalling. Contrary to previous reports, we found that dimers are not disulfide linked constitutively or following ICAM-1 ligation, and that membrane-proximal cysteine residues are not required for dimerization or ICAM-1 induced events.

## Materials and methods

### Antibodies and Reagents

CT2 Armenian Hamster monoclonal antibody (mAb) [20], directed against the last 17 C-terminal amino acids of MUC1-CD, was generously provided by Dr. Sandra Gendler (Mayo Clinic, Scottsdale, AZ). Rabbit anti-Src mAb, anti-Src^P416 ^polyclonal Abs, and anti-rabbit peroxidase conjugated secondary antibody were purchased from Cell Signalling. Goat anti-mouse and anti-Armenian hamster peroxidase-conjugated secondary antibodies were purchased from Jackson ImmunoResearch Laboratories, Inc. Mouse anti-tubulin antibody was from Sigma-Aldrich. Disuccinimidyl suberate (DSS) was from PierceNet. Protein G-Agarose was purchased from Roche Diagnostics. ECL Plus Western Blotting detection reagent was purchased from GE Healthcare (Amersham Biosciences). Gelatin Type A and phosphatase inhibitor cocktail were from Sigma-Aldrich. Protease inhibitor cocktail was from Calbiochem. Dulbecco's modified eagle media (DMEM), fetal bovine serum (FBS), Lipofectamine 2000, G418, Blasticidin S HCl, Pluronic F-127, Fluo-3 and Cell Tracker Green CMFDA were from Invitrogen. AP20187^D ^and AP21998^M ^were generous gifts from ARIAD Pharmaceuticals, Inc (Cambridge, MA, USA).

### Plasmid construction

The pC1-Neo-hMUC1-TR+ plasmid was kindly provided by Dr. Sandra Gendler. The pUC-CVM-MUCY plasmid was from Gene-Therapeutics Luckenwalde (Luckenwalde, Germany). MUC1-CFP and MUCY-YFP were constructed by inserting the MUC1/MUCY genes into pECFP/pEYFP plasmids (ClonTech) respectively, at XhoI and SacII sites. The plasmid pC4-Fv1E encoding the FKBP F36V variant followed by a c-terminal hemagglutinin (HA) epitope was generously provided by ARIAD Pharmaceuticals Inc. To generate the MUC1-CFP-FvHA and MUCY-YFP-FvHA fusion proteins, the FvHA domain of pC4-Fv1E was amplified by polymerase chain reaction (PCR) with a 5' primer (ATTTGTACATGGCTTCTAGAGGAGTGC) and a 3' primer (CTCTTGTACACTGAAGTTCTCAGGATCC) which introduced 3' and 5' BsrG1 restriction sites (underlined). The PCR product and MUC1-CFP/MUCY-YFP plasmids were digested with BsrG1, ligated, and sequenced to confirm insertion and orientation. MUC1-CFP-FvHA (CQC to AQA) was constructed by PCR of MUC1-CFP using overlapping forward (TTGGCTGTCGCTCAGGCCCGCCGAAAG) and reverse (CTTTCGGCGGGCCTGAGCGACAGCCAA) primers to generate the mutation (underlined) and upstream (GGCACCTCTGCCAGGGCTACCACAACC) and downstream (GACCGGTGGATCCCGGGCCCG) primers containing EcoN1 and BamH1 restriction sites, respectively (underlined). Digestion of plasmid and PCR product with EcoN1 and BamH1 was followed by ligation of the plasmid backbone and mutated insert and sequencing to confirm insert. The pcDNA3.1-CD8/MUC1 plasmid was kindly provided by Dr. K.C. Kim (Lovelace Respiratory Research Institute, AZ), and encodes a construct containing the extracellular and transmembrane portions of cluster of differentiation 8 (CD8) and MUC1-CD, beginning at R^4^RK (Figure 1).

### Cell culture

Human breast cancer cell lines T47D and MCF-7 were from the American Type Culture Collection (ATCC) and were maintained in DMEM with 10% FBS and 6 ug/ml insulin. 293T human embryonic kidney epithelial cells (293T HEK) were from ATCC and maintained in DMEM with 10% FBS. Mock and ICAM-1 transfected NIH 3T3 mouse fibroblast cells were a generous gift of Dr. Ken Dimock (University of Ottawa, Ontario, Canada) and were maintained in DMEM with 10% FBS and 5 ug/ml Blasticidin S. HEK 293T cells transfected with MUC1 constructs were maintained in DMEM with 10% FBS and 200 ug/ml G418 and used for experiments within 48 hours of transfection. Cell lines have not been further tested or authenticated.

### Small interfering ribonucleic acid (siRNA) knockdown

4 × 10^5 ^HEK 293T cells were plated in a 6-well plate and allowed to adhere overnight to approximately 50% confluency. siRNA (Dharmacon) consisted of four pooled siRNA species targeting the following Src sequences: GCAGUUGUAUGCUGUGGUU, GCAGAGAACCCGA GAGGGA, CCAAGGGCCUCA ACGUGAA, and GGGAGAACCUCUAGGCACA. Transfection was performed using Lipofectamine 2000 (Invitrogen), according to the manufacturer's instructions. Lipofectamine reagent only or non-targeting siRNA were used as negative controls.

### Dimer detection

To detect constitutive dimers, we added a cross-linking agent before lysis to parallel cultures and analyzed by Western blot as follows. 3 × 10^6 ^human breast cancer cells or transfected HEK 293T cells were plated on 0.1% gelatin coated, UV-treated 10 cm dishes and allowed to adhere overnight. Cells were then serum starved for 45 minutes in Imaging Buffer (152 mM NaCl, 5.4 mM KCl, 0.8 mM MgCl_2 _6H_2_O, 1.8 mM CaCl_2 _2H_2_O, 10 mM HEPES, 5.6 mM D-glucose). Treatment compounds or cell suspensions were then added as indicated, in 37°C Imaging buffer, followed by 1 mM DSS in ice-cold PBS for 10 minutes. DSS is a membrane-permeable, permanent crosslinker which targets primary amine residues (lysines) within 11.4 Å of eachother. DSS does not induce dimerization of lysine-containing proteins, but rather aids in identification of protein complexes which are already formed upon treatment. DSS was aspirated, cells resuspended in quenching solution (1M Tris, pH 7.5) and centrifuged. The pellet was suspended in ice-cold lysis buffer (20 mM Tris-HCl pH 7.4, 150 mM NaCl, 2 mM EDTA, 1% glycerol, 1% Triton X-100, 0.5% phosphatase and protease inhibitors) or Co-immunoprecipitation lysis buffer (50 mM Tris pH 7.6, 100 mM NaCl, 0.5 mM EDTA, 0.5% Nonidet P-40, 0.5% protease and phosphatase inhibitors). Lysates were immunoprecipitated with CT2 and/or prepared for Western blot analysis as described below.

### Dimer manipulation

To artificially manipulate MUC1-CD dimerization we used the "ARGENT™ Regulated homodimerization kit" and the "RPD™ Regulated secretion/aggregation kit" (Ariad Pharmacauticals, Inc.). The kits were designed to manipulate protein dimerization status by interacting an engineered "Fv dimerization domain" with monovalent and bivalent ligands. The "Fv domain" is a mutant of the naturally occurring FK506 binding protein (FKBP) with a F36V mutation introduced to prevent binding of Fv ligands to endogenous FKBP. A MUC1-CFP-Fv construct was creating using this plasmid, as described above. Importantly, the Fv domain itself does not induce dimerization, and addition of Fv domain ligands is required to manipulate dimerization status of Fv domain containing proteins. The bivalent Fv ligand AP20187^D ^was designed to induce dimerization of Fv domain containing proteins, while the monovalent Fv ligand AP21998^M ^was designed to disaggregate existing dimers.

### Immunoprecipitation and Western blots

Lysates were immunoprecipitated, prepared for SDS-PAGE, and probed for proteins of interest as described in [6]. Films were scanned with a Canon Canoscan 8600F, imported into Image J (NIH), contrast and brightness adjusted and cropped for presentation.

### Calcium oscillation assay

Calcium oscillation assay was performed and analyzed as described [5]. Modifications included the treatment of cells with 1 uM AP21998^M ^or AP20187^D ^for 1 minute prior to addition of NIH ICAM-1 cells, and the use of Eclipse software to obtain digital interference contrast (DIC) and fluorescent images.

### Transwell migration assay

The upper membrane of Transwell inserts (Corning Costar, 6.5 mm diameter, 8 μm pore size) coated with 0.1% gelatin and 200 ul of ICAM-1/mock cell suspension at 1.5 × 10^5 ^cells/ml was placed in a 24-well plate and allowed to adhere overnight at 37°C. 293T MUC1 transfectants were suspended in 5 uM Cell tracker green in serum-free DMEM for 30 minutes at 37°C, followed by incubation in serum free DMEM at 37°C for 30 minutes. Cells were spun and suspended in serum-free media at 8 × 10^5 ^cells/ml. 1 uM AP21998^M ^or AP20187^D ^was added and 200 ul of cell suspension was added to the upper membrane of Transwell inserts. Fresh serum-free DMEM was added to the lower chamber. Following incubation at 37°C overnight, media was removed and 2% paraformaldehyde in PBS was added to each chamber for 15 minutes. Cells were washed twice with PBS, and cells on the upper membrane of the insert were removed with a sterile cotton swab. The insert was then placed under a Zeiss Axioscope Digital Imaging Microscope and cells on the lower side of the chamber were counted under a fluorescein isothiocyanate filter and 20× objective for five distinct fields of view.

### Statistics

All experiments were performed at least three times to allow for statistical analysis. The Newman-Keuls multiple range comparison was used to determine statistical differences in data sets with more than two experimental conditions. For pairwise comparisons, the Student's *t *test was used. P values are indicated for each analysis. For each experiment, pairs in the data set which are statistically different, or populations which do not overlap with any other in the data set, (p < 0.05) are indicated with an *asterisk *(*).

## Results

### MUC1/ICAM-1 binding induced signalling is mediated by Src kinase

**Figure 2:**
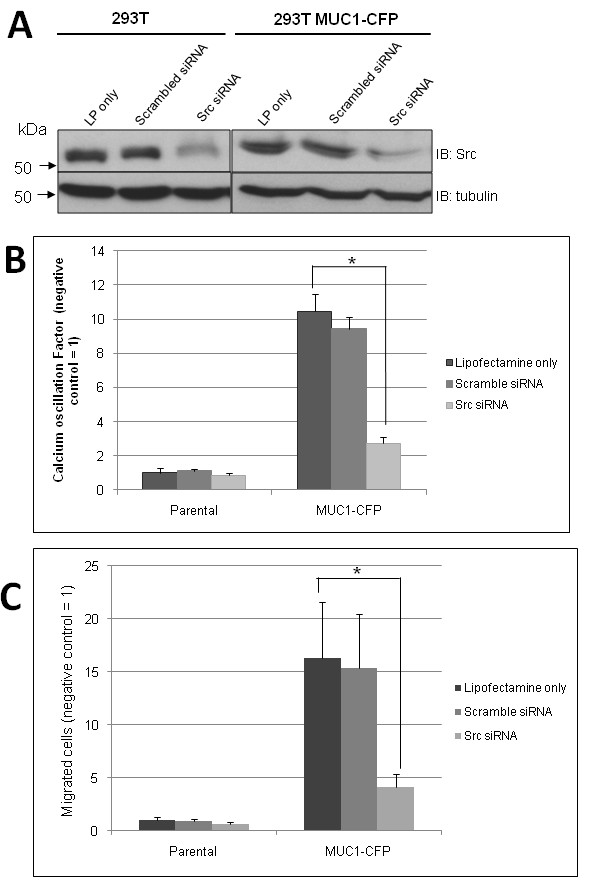
**Src knockdown and ICAM-1 induced signalling**. **A**. HEK 293T cells without (left panel) or with (right panel) MUC1-CFP expression were transfected with Scramble or Src targeted siRNA using Lipofectamine 2000. Following lysis and SDS-PAGE, blots were probed for Src, or as a control, tubulin. As a control, cells were treated with Lipofectamine 2000 reagent only (LP only). Cells were then assayed for ICAM-1 binding induced calcium oscillations (**B**) or migration through an ICAM-1 positive monolayer (**C**). HEK 293T, LP only condition was set to one and remaining conditions expressed as a ratio. Columns represent average oscillation factor (**B**) or average number of migrated cells per five fields (**C**) from at least three independent trials; bars, SE. *Asterisk *indicates pairs in the data set which are statistically different (p < 0.05).

We first confirmed that Src kinase is a critical component of the MUC1/ICAM-1 signalling axis by siRNA knockdown of Src in MUC1-CFP transfected HEK 293T cells. After Src siRNA treatment, we obtained a ~50% reduction in the levels of Src protein, compared to treatment with Lipofectamine alone or scrambled siRNA (Figure 2a). We then assayed for calcium oscillations (Figure 2b) and migration (Figure 2c), and found that MUC1-CFP cells treated with Lipofectamine-only respond to ICAM-1 stimulation by generating calcium oscillations (Figure 2b) and cell migration (Figure 2c), indicating that the presence of the CFP tail does not interfere with this response. Non-transfected HEK 293T cells which have no MUC1 showed no difference in ICAM-1 binding induced calcium oscillations or migration with decreased Src. Addition of scrambled siRNA to the transfected MUC1-CFP cells showed no decrease in Src levels and levels of ICAM-1 binding induced events equivalent to the Lipofectamine-only condition (negative control). However, Src siRNA induced Src knockdown in HEK 293T cells transfected with MUC1-CFP resulted in significant decreases in the ICAM-1 binding initiated calcium oscillations (Figure 2b) and transmigration through an ICAM-1 monolayer (Figure 2c). This establishs Src kinase as an essential mediator of MUC1/ICAM-1 binding signalling and migration.

### MUC1 forms constitutive cytoplasmic domain dimers in human breast cancer cell lines and transfected HEK 293T cells

**Figure 3:**
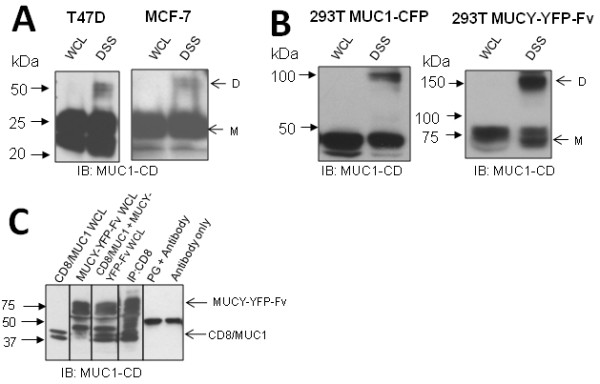
**MUC1 dimerization in human breast cancer and transfected cell lines**. **A**. Breast cancer cell lines T47D and MCF-7, or (**B) **HEK 293T cells transfected with MUC1 constructs MUC1-CFP and MUCY-YFP-Fv were treated with DSS or no treatment control, lysed, ran on SDS-PAGE and probed with anti-MUC1-CD. **C**. HEK 293T cells were transfected with CD8/MUC1 (lane 1) MUCY-YFP-Fv (lane 2), or both (lane 3), lysed, and double transfectants immunoprecipitated with anti-CD8 (lane 4). Lysates were ran on SDS-PAGE and probed with anti-MUC1-CD. "D" and "M" indicate expected molecular weights of dimer and monomer, respectively. Whole cell lysates (WCL), PG (Protein G) + Antibody (anti-CD8) and Antibody only (Anti-CD8) lanes are included as controls.

MUC1 positive human breast cancer cell lines MCF-7 and T47D (Figure 3a) and HEK 293T cells transfected with MUC1-CFP (Figure 3b, panel 1) or the MUC1 splice variant lacking the tandem repeat domain MUCY-YFP-Fv (Figure 3b, panel 2) were lysed with or without prior treatment with the membrane permeable crosslinker DSS. No Fv ligands were added, so only constitutive dimers of the MUCY-YFP-Fv are detectable. DSS reacts with primary amine containing amino acids within 11.4Å distance to produce covalently bonded complexes. It is important to note that DSS only reacts with lysine residues which are within 11.4 Å of each other prior to DSS treatment; DSS itself does not induce complex formation. MUC1-CD contains a membrane-proximal lysine residue (R^4^RK) which would be susceptible to DSS crosslinking. Western blotting and probing for MUC1-CD in the cells treated with DSS revealed the invariable appearance of a new species at exactly double the molecular weight of the monomeric cytoplasmic domain, consistent with the presence of a MUC1-CD homodimer. The appearance of MUC1-CD dimers in MUCY-YFP-Fv transfectants indicates that the tandem repeat domain is not responsible for dimerization. This is not surprising due to the heavy glycosylation and negative charge of the tandem repeats. We then investigated the contribution of the MUC1 cytoplasmic domain to dimer formation. HEK 293T cells were co-transfected with MUCY-YFP-Fv and/or CD8/MUC1 [21], a chimera of CD8 extracellular and transmembrane domains and MUC1-CD domain, beginning at R^4^RK (Figure 1). Whole cell lysate of CD8/MUC1 (Figure 3c, lane 1) shows that this construct appears as a doublet at approximately 40 kDa in agreement with a publication describing this construct [21]. MUCY-YFP-Fv runs at approximately 75 kDa (Figure 3c, lane 2), with another species migrating at approximately 45 kDa. This species could be the result of cleavage of the YFP-Fv tag prior to cell lysis, as MUCY is expected to migrate at this molecular weight. Dual transfection of CD8/MUC1 and MUCY-YFP-Fv demonstrates that both these constructs run at the expected molecular weights when co-expressed (Figure 3c, lane 3). Immunoprecipitation of double transfectants with anti-CD8 resulted in the appearance of MUCY-YFP-Fv on a Western blot (Figure 3c, lane 4), indicating an association between CD8/MUC1 and MUCY-YFP-Fv. This association is significant because the CD8/MUC1 construct only contains the cytoplasmic portion of MUC1, beginning at R^4^RK and does not contain the membrane proximal C^1^QC motif, fluorescent tags or an Fv domain. Therefore association between these two entities must be due to the MUC1 cytoplasmic domain. "Protein G + Antibody" and "Antibody only" lanes are included to identify non-specific immunoglobulin bands. Taken together, these data indicate that the cytoplasmic domain of MUC1 self-associates to form a constitutive homodimer.

### MUC1-CD dimerization is independent of membrane-proximal cysteine residues

**Figure 4:**
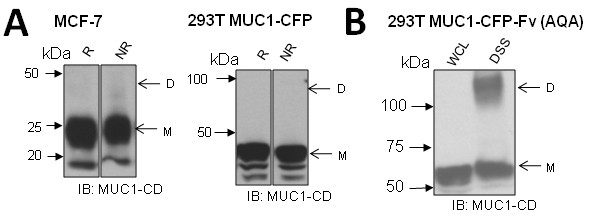
**Covalent bonds and cysteine resides in MUC1 dimerization**. **A**. Breast cancer cell line MCF-7 or HEK 293T cells transfected with MUC1-CFP were subjected to reducing ("R"; β-mercaptoethanol added) or non-reducing ("NR"; no β-mercaptoethanol) conditions, ran on separate SDS-PAGE to prevent diffusion of β-mercaptoethanol, and probed with anti-MUC1-CD. **B**. 293T MUC1-CFP-Fv AQA cells were treated with DSS, lysed, ran on SDS-PAGE, and probed with anti-MUC1-CD. "D" and "M" indicate expected molecular weights of dimer and monomer, respectively. Whole cell lysates (WCL) are included as controls.

Previous publications investigating MUC1 dimerization have concluded that the membrane-proximal CQC motif is responsible for disulfide-linked oligomerization, which results in targeting of MUC1 to the nucleus [18], and MUC1 mediated resistance to oxidative stress [19,22,23]. Since the CD8/MUC1-MUCY co-immunoprecipitation experiments (Figure 3c) found MUC1-CD association in the absence of a CQC motif in the CD8/MUC1 partner, we sought to determine if CQC mediated dimerization was necessary for our observed constitutive MUC1 dimers in MUC1 full-length transfectants and breast cancer cell lines. Using non-reducing conditions, which would preserve any disulfide linkages in Western blotting, there were no bands at a molecular weight of presumed dimers in 293T MUC1-CFP or MCF-7 cells (Figure 4a). This suggests that MUC1-CD dimers are not disulfide linked. We confirmed this by mutating both cysteines in the native MUC1-CD CQC motif to alanines (AQA) and then assaying for dimers after DSS cross-linking. Note that no Fv ligands were added so that any dimers detected represent constitutive or pre-formed dimers. We found that the 293T MUC1-CFP-Fv (AQA) mutant formed cytoplasmic domain dimers (Figure 4b). The presence of DSS-stabilized dimers in the absence of Fv ligands indicates that constitutive MUC1-CD dimers form even when both cysteines are absent and constitutes conclusive proof that cellular MUC1-CD dimers are not disulfide linked.

### MUC1 cytoplasmic domain dimerization can be disrupted by an engineered Fv domain and a monomeric ligand

**Figure 5:**
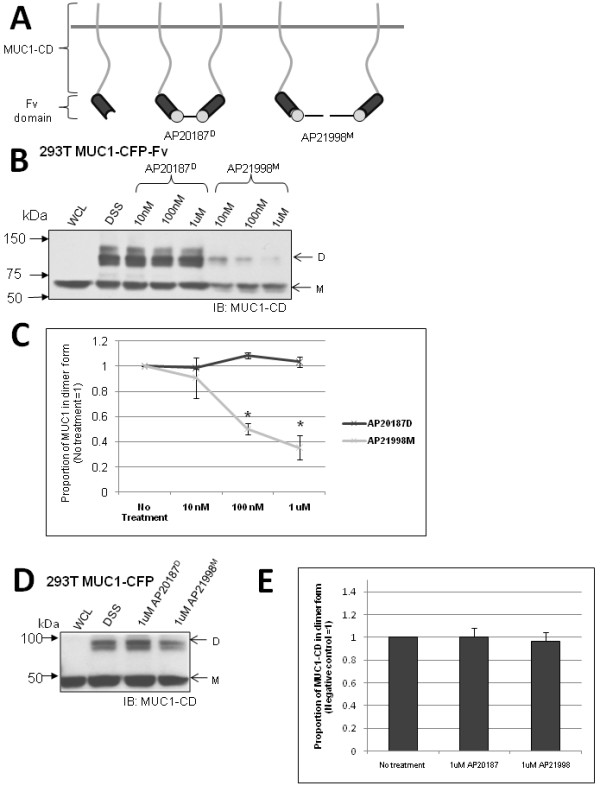
**Manipulation of MUC1 dimerization using an engineered domain**. **A**. Schematic of the mechanism of dimer formation/disruption by Fv ligands. **B**. Treatment of 293T MUC1-CFP-Fv cells with AP20187^D^/AP21998^M ^and DSS. **C**. Densitometric analysis of dimer bands from **B **normalized to total MUC1. "No treatment" control refers to "DSS" only treatment from (**B**), and it set to one with the remaining conditions expressed as a ratio. **D**. Treatment of 293T MUC1-CFP cells with AP20187^D^/AP21998^M ^and DSS. **E**. Densitometry of MUC1-CFP dimer bands from **D **normalized to total MUC1. "No treatment" control refers to "DSS" only treatment from (**D**) and is set to one with the remaining conditions expressed as a ratio. "D" and "M" indicate expected molecular weights of dimer and monomer, respectively. Whole cell lysates (WCL) are included as controls. *Asterisk *indicates a discrete population that does not overlap with any other population in the data set, p < 0.05.

To investigate the importance of MUC1 dimerization in Src association and ICAM-1 induced signalling, we manipulated dimerization using a chimeric construct of MUC1 and a C-terminal Fv domain (ARIAD Pharmaceuticals), which is FKBP (FK506 binding protein) with a F36V mutation, allowing for specific interaction between the engineered Fv domain and bivalent (AP20187^D^) or monovalent (AP21998^M^) ligands. Importantly, the Fv domain itself does not facilitate dimerization of proteins, but following addition of Fv domain ligands, dimerization status can be manipulated. Previously, this system has been used to successfully manipulate dimerization of growth factor receptors [24] and G protein-coupled receptors [25]. Mechanistically, the bivalent ligand, which contains two Fv-binding domains, effectively brings two Fv-domain containing proteins within close proximity - "dimerization". The monovalent ligand, which contains one Fv-domain binding domain, is designed to bind to Fv-domain containing proteins and sterically inhibit their interaction with other proteins - "disaggregation" or "monomerization" (Figure 5a). MUC1-CD dimers were stabilized after Fv ligand treatment by addition of the DSS cross-linker prior to cell lysis. We found that treatment of 293T MUC1-CFP-Fv cells for one minute with increasing concentrations of AP20187^D ^did not increase the quantity of MUC1-CD dimers above no treatment, while AP21998^M ^treatment resulted in a dose dependant reduction in MUC1-CD dimerization (Figure 5b). After treatment with 1 μM of monomerizing Fv ligand, AP21998^M ^there was a 60% reduction in detectable MUC1-CD dimers (Figure 5c). As a control, 293T MUC1-CFP cells, which lack the Fv domain, do not show a change in dimer quantity following treatment with AP20187^D ^or AP21998^M ^(Figure 5d). Densitometric analysis of the MUC1-CFP dimer band normalized to monomer band illustrates this observation further (Figure 5e).

### MUC1-CD dimer disruption results in decreased recruitment of total and active Src kinase

**Figure 6:**
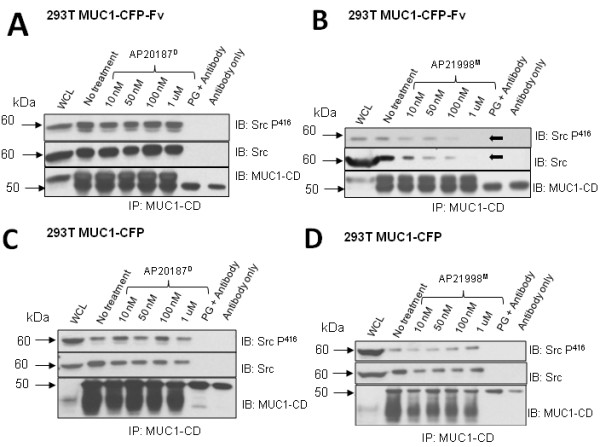
**Src and Src^P416 ^recruitment to MUC1 after manipulation of dimerization**. Co-immunoprecipitation with anti-MUC1-CD was performed on 293T MUC1-CFP-Fv cells **(A, B) **and MUC1-CFP cells **(C, D) **after treatment with increasing concentrations of AP20187^D ^**(A, C) **or AP21998^M ^**(B, D)**. Immunoprecipitates were probed for Src^P416^, Src, and anti-MUC1-CD (as a loading control) with stripping of blots between each probe. Whole cell lysates (WCL), PG (Protein G) + Antibody and Antibody only lanes are included as controls. Densitometric analysis of Src and Src^P416 ^compared to MUC1-CD is presented in Additional File 1.

To determine the importance of MUC1-CD dimerization in constitutive Src recruitment, 293T MUC1-CFP-Fv (Figure 6a, b), and, as a control, 293T MUC1-CFP (Figure 6c, d) cells were treated with increasing concentrations of AP20187^D ^or AP21998^M ^for one minute and immunoprecipitated with anti-MUC1-CD. Following separation on SDS-PAGE, blots were probed with anti-Src (total) and anti-Src^P416 ^(active). In the MUC1-CFP-Fv transfectants, the amount of total and active Src associated with MUC1-CD decreases in a dose-dependent manner with AP21998^M ^treatment (Figure 6b, arrows). Treatment with AP20187^D ^did not result in a significant change, and 293T MUC1-CFP cells were unaffected by Fv ligand treatment.

Densitometric analysis of Src and Src^P416 ^compared to MUC1-CD are given in Additional File 1. These data suggest that MUC1-CD dimers, but not monomers, contain a recruitment, and potentially an activation, motif for Src kinase.

### MUC1-CD dimer disruption results in decreased ICAM-1 binding induced calcium oscillations and cell invasion

**Figure 7:**
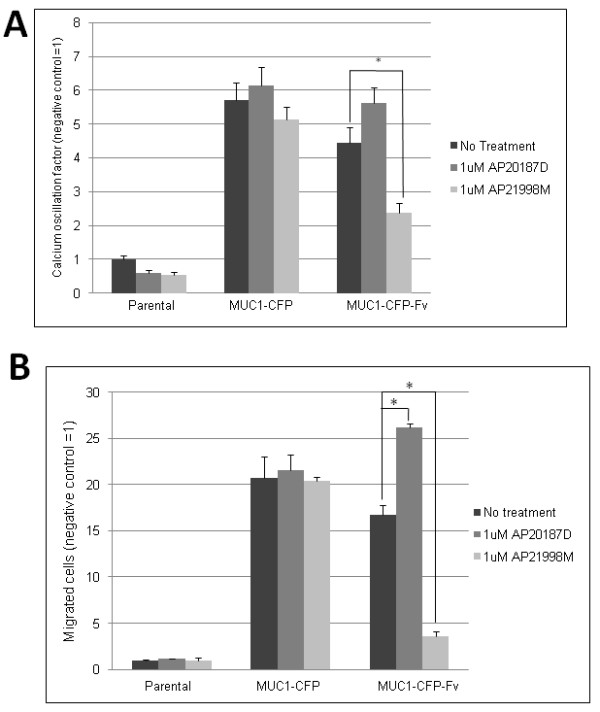
**ICAM-1 induced signalling in MUC1 cells after manipulation of dimerization**. HEK 293T (parental), MUC1-CFP, and MUC1-CFP-Fv cells were assayed for (**A**) ICAM-1 binding induced calcium oscillations or (**B**) migration through an ICAM-1 positive monolayer, after No treatment, 1 uM AP20187^D^, or 1 uM AP21998^M^. HEK 293T (parental), no treatment condition was set to one and remaining conditions expressed as a ratio. Columns represent average oscillation factor (**A**) or average number of migrated cells per five fields (**B**) from at least three independent trials; bars, SE. *Asterisk *indicates pairs in the data set which are statistically different (p < 0.05).

**Figure 8:**
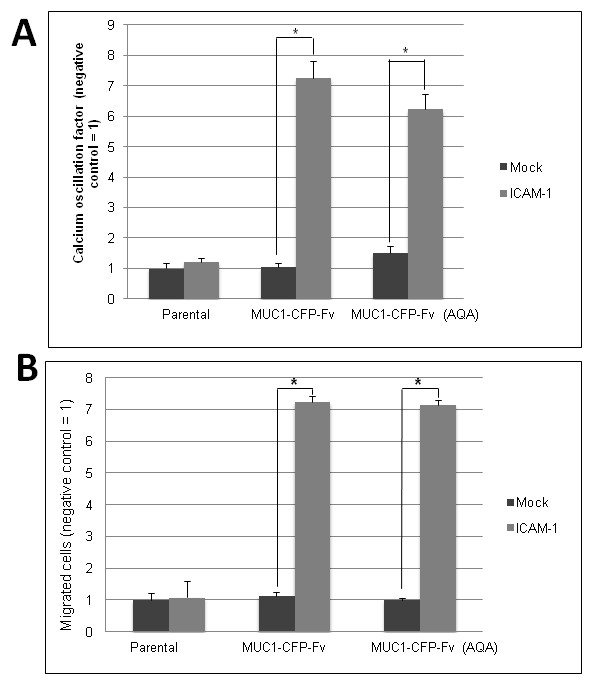
**ICAM-1 induced signalling in MUC1-CFP-Fv (AQA) transfected cells**. HEK 293T (parental), MUC1-CFP-Fv and MUC1-CFP-Fv (AQA) cells were assayed for (**A**) ICAM-1 binding induced calcium oscillations and (**B**) migration through an ICAM-1 positive monolayer. HEK 293T (parental), mock (no ICAM-1) condition was set to one and remaining conditions expressed as a ratio. Columns represent average oscillation factor (**A**) or average number of migrated cells per five fields (**B**) from at least three independent trials; bars, SE. *Asterisk *indicates pairs in the data set which are statistically different (p < 0.05).

To determine if MUC1-CD dimerization is important in previously observed ICAM-1 binding induced events, we assayed parental (293T), 293T MUC1-CFP, and 293T MUC1-CFP-Fv, and 293T MUC1-CFP-Fv (AQA) cells for ICAM-1 binding induced calcium oscillations, and invasion through an ICAM-1 positive monolayer after addition of the Fv ligands 1 μM AP20187^D ^or 1 μM AP21998^M ^and compared this to a no treatment control. 293T MUC1-CFP and 293T MUC1-CFP-Fv cells responded to treatment with ICAM-1 positive cells, in the "no treatment" condition, by initiating calcium oscillations (Figure 7a) and increased invasion (Figure 7b) at levels which were significantly increased compared to Mock treatment and statistically equivalent, demonstrating that the addition of the CFP or CFP-Fv tag on the C-terminus did not affect MUC1 receptor response to ICAM-1 stimulation. The 293T MUC1-CFP-Fv (AQA) mutant also exhibited ICAM-1 binding induced calcium oscillations (Figure 8a) and cell invasion (Figure 8b) equivalent to the native 293T MUC1-CFP-Fv cells indicating that the CQC motif is not required for ICAM-1 induced signalling events. However, ICAM-1 binding induced calcium oscillations (Figure 7a) and invasion (Figure 7b) in 293T MUC1-CFP-Fv cells was significantly reduced by treatment with AP21998^M^, while prolonged treatment with AP20187^D ^resulted in a significant increase in cell migration in 293T MUC1-CFP-Fv cells (Figure 7b). These data indicate that MUC1-CD dimerization is required for ICAM-1 binding induced events. Addition of the Fv ligands had no significant effect on the 293T MUC1-CFP transfectants lacking the Fv domain. As previous reports [19] have demonstrated that disruption of dimerization using peptides results in cell death, we performed a trypan blue exclusion assay after treatment with 1 μM AP20187^D ^or AP21998^M ^and saw no significant reduction in viability up to 72 hour exposure (See Additional File 2).

### ICAM-1 ligation does not result in increased MUC1-CD dimerization or disulfide linkage of MUC1-CD dimers

**Figure 9:**
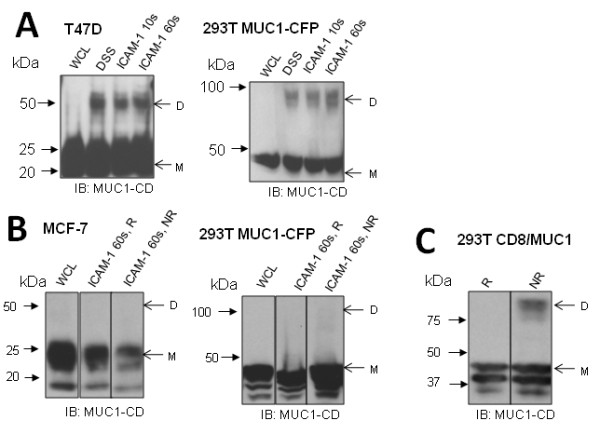
**Covalent dimerization of MUC1 following ICAM-1 binding**. **A**. Breast cancer cell line T47D and 293T-MUC1-CFP cells were stimulated with NIH-ICAM-1 cells for 10 s or 60 s and treated with DSS, lysed, ran on SDS-PAGE and probed with anti-MUC1-CD. **B**. Breast cancer cell line MCF-7 and 293T MUC1-CFP cells were lysed with or without prior treatment with NIH ICAM-1 cells for 60 seconds. Lysates were subjected to reducing ("R"; β-mercaptoethanol added) or non-reducing ("NR"; no β-mercaptoethanol) conditions, ran on separate SDS-PAGE to prevent diffusion of β-mercaptoethanol and probed with anti-MUC1-CD. **C**. 293T CD8/MUC1 cell lysate was run under reducing or non-reducing conditions, ran on separate SDS-PAGE to prevent diffusion of β-mercaptoethanol and probed with anti-MUC1-CD. "D" and "M" indicate expected molecular weights of dimer and monomer, respectively. Whole cell lysates (WCL) are included as controls.

Next, we investigated if ICAM-1 ligation results in a quantitative increase in MUC1 dimerization, a potential mechanism for signal initiation. T47D and 293T MUC1-CFP cells were treated with ICAM-1 transfected NIH 3T3 cells for 10 or 60 seconds prior to DSS treatment. These time points were chosen because previous work has demonstrated that increased Src and CrkL recruitment, MUC1-CD phosphorylation [6] and calcium oscillations [5] occur within one minute of ICAM-1 ligation. We found that ICAM-1 ligation did not increase the quantity of MUC1-CD dimer detected (Figure 9a), suggesting that a qualitative, rather than quantitative, change in MUC1-CD dimers is responsible for ICAM-1 induced signalling. We then considered the possibility that ICAM-1 binding triggers disulfide bridge formation, akin to growth hormone binding to pre-formed growth hormone receptor dimers [15], but were not able to detect MUC1-CD dimers in MCF-7 or 293T-MUC1-CFP cells subjected to reducing or non-reducing conditions after ICAM-1 treatment for 60 seconds (Figure 9b). As a control, the CD8/MUC1 chimera, which is disulfide linked via the CD8 extracellular region [26,27], was run under reducing and non-reducing conditions. The appearance of a disulfide-linked dimer under non-reducing conditions (Figure 9c) validates our methods.

## Discussion

The MUC1 glycoprotein has been implicated in multiple tumorigenic processes including tumour formation, proliferation, and survival [19,22,28,29]. We are unique in investigating the role of MUC1 in the motility of Luminal B breast cancer cell lines, focusing on the binding of MUC1 to ICAM-1. ICAM-1 is expressed throughout the migratory track of a metastasizing breast cancer cell and its role in leukocyte extravasation is well characterized [30,31]. Although we have previously shown that MUC1/ICAM-1 ligation induces pro-motility behaviour [5-7], the mechanism of signal initiation was unknown. In this report, we show that dimerization of the MUC1-CD is essential for the ICAM-1 induced events and that this effect is most likely mediated through enhanced Src binding.

The signalling capacity of transmembrane proteins lacking kinase activity is often mediated by associated non-receptor tyrosine kinases. Here we show that Src kinase is essential for transmission of the migration related MUC1/ICAM-1 signal. This is consistent with the literature on Src inhibition in breast cancer. Even though transfection of Src alone does not have transforming ability [32], over activity of Src is commonly associated with breast tumour progression [33] and it has become a prime target for selective small molecule inhibitors: Dasatinib (Bristol-Myers Squibb), Bosutinib (Wyeth) and Saracatinib (AstraZeneca). Others have published that the MCF-7 luminal B cell line used in this study shows decreased migration when Src is inhibited [34-36]. This decrease in Src mediated cell migration is synergistic with concomitant Tamoxifen [37], associated with upregulation and stabilization of E- cadherin/β-catenin mediated intercellular adhesion [36,38], and decreased activity of the integrin associated kinase, focal adhesion kinase (FAK) [34,37]. These observations are consistent with MUC1 involvement in the observed Src motility pathway. Tamoxifen decreases MUC1 expression [39,40] and down-regulation of MUC1 is associated with increased E-cadherin/β-catenin complex formation [41]. Further, MUC1 has been shown to bind to FAK, possibly transporting Src to FAK forming a MUC1-Src-FAK complex, and increasing FAK activation [28]. Thus the additive effect of Tamoxifen, the stabilization of intercellular adhesions and the decreased FAK activity are logical consequences of the dual inhibition of MUC1 and Src in the same pathway.

The association between MUC1 and Src is dependent on the existence of MUC1-CD dimers, indicating that MUC1-CD dimers adopt a conformation that is permissive Src binding. We have definitive (unpublished) data demonstrating that a Src-SH3 peptide binds to MUC1 constitutively via the putative SH3 binding domain R^34^XXP^37^P^38^XXXXR^43^. Binding of the Src SH3 domain has been previously described as a mechanism for partial unfolding of the inactive Src enzyme and can be associated with Src activation [42] suggesting a mechanism for MUC1-CD dimer activation of Src. In this regard, it is significant that Src-P^416^, which is indicative of fully active Src, also selectively binds to MUC1-CD dimers.

Classically, it was believed that surface membrane receptors existed as monomers until ligand binding induced dimerization of the receptors, allowing trans-activation of receptor associated kinases and the triggering of signal initiating phosphorylation cascades. In recent years, a new paradigm, typified by the growth hormone receptor (GHR), has emerged in which receptors exist as pre-formed ligand-independent dimers [[13], Reviewed in [14]]. Upon ligand binding to the dimers, structural changes such as cysteine linkage, association with detergent resistant membrane fractions or changes in receptor cleavage result in signal initiation [15-17]. We report here that the MUC1 cytoplasmic domain exists constitutively as a non-covalently linked dimer. We present evidence that in the absence of the transmembrane and extracellular domains, the cytoplasmic domain of MUC1 self-associates in a non-cysteine dependent fashion. It has been proposed that a "self-aggregation domain" exists in the extracellular stub of MUC1-CD [43], but further studies are required to address the possibility that functional dimerization in vivo involves several domains.

In this study, membrane proximal cysteine residues are not required for dimerization of MUC1-CD, Src recruitment, or ICAM-1 induced signalling indicating that disulfide bridge formation is not the ligand-induced signal initiating event, as has been proposed for GHR. This is to be expected since, in the reducing environment of the cytosol, formation and maintenance of disulfide bonds is unfavourable unless the redox balance is disrupted [44]. Thus, disulfide linkage of MUC1 dimers reported by others [18,19], represents an alternative functional pathway for MUC1-CD dimers, perhaps as a redox "sensor" [Reviewed in [45]], that is unrelated to the ICAM-1/Src motility pathway in the present study. In this way, cysteine-mediated dimerization of MUC1 in response to oxidative stress, could initiate a signalling cascade resulting in the demonstrated nuclear entry and expression of anti-oxidant enzymes ascribed to cysteine-linked MUC1-CD dimers [19,22,23].

Rational drug combination has received considerable interest in recent years [Reviewed in [46]] as it provides the opportunity for specific, synergistic inhibition of cell signalling pathways. Initial clinical results using Src inhibitors as single agents has shown them to be well-tolerated but have minimal anti-tumour response in patients [47]. Several Src inhibitors are currently undergoing testing in clinical trials for use in breast cancer treatment alone and in combination with other inhibitors [Reviewed in [48]]. The subset of luminal B cancers with active Src kinase pathway [49] may be the ideal target for a combined Tamoxifen and anti-Src therapy. Our studies suggest that if these could be combined with an inhibitor of MUC1 dimerization that cell migration and metastases may be significantly decreased, possibly without the toxic effects of classic chemotherapy.

## Conclusion

The MUC1 protein is overexpressed in the majority of breast cancers and is implicated in breast cancer metastasis. We show here for the first time that MUC1-CD forms non-covalently linked dimers which are required for recruitment of Src kinase, and ICAM-1 induced pro-metastatic events. This is significant because ICAM-1 is expressed throughout the migratory tract of a metastasizing breast cancer cell. Ligation of MUC1 and ICAM-1 may represent a mechanism for movement of breast cancer cells through stromal and endothelial tissues. Therefore, elucidation of the mechanism of MUC1/ICAM-1 signalling will reveal potential targets for anti-metastatic therapies. Our study sheds light on this mechanism and also demonstrates the need for additional research to resolve discrepancies in the field.

## List of Abbreviations

Å: angstrom; aa: amino acid; Ab: antibody; ATCC: American tissue culture collection; CD8: cluster of differentiation 8; DIC: digital interference contrast; DMEM- Dulbecco's modified eagle media; DSS: disuccinimidyl suberate; FAK: focal adhesion kinase; FBS: fetal bovine serum; FKBP: FK506 binding protein; GHR: growth hormone receptor; HA: hemagglutinin; HEK: human embryonic kidney; ICAM-1: Intercellular adhesion molecule-1; MUC1: Mucin-1; MUC1-CD: Mucin-1 cytoplasmic domain; PBS: phosphate buffered saline; PCR: polymerase chain reaction; SDS-PAGE-Sodium dodecyl sulphate-polyacrylamide gel electrophoresis; siRNA: small interfering ribonucleic acid.

## Competing interests

The authors declare that they have no competing interests.

## Authors' contributions

AB contributed to study design, performed all experiments, analyzed data, contributed to data interpretation, and drafted the manuscript. JZ contributed to study design and constructed plasmids. EL contributed the MUC1/CD8 plasmid and contributed to data interpretation. AS contributed to study design and data interpretation. NG performed complimentary studies that aided in the interpretation of this data. JH conceived the study, contributed to study design, and helped to draft and edit the manuscript. All authors read and approved the final manuscript.

## Supplementary Material

Additional file 1 **Densitometry of Src and Src^P416 ^bands from SDS-PAGE (Figure **6**) normalized to MUC1-CD**. Using ImageJ software (NIH), the Src and Src ^P416 ^bands were analyzed for densitometric intensity, and values were normalized to the intensity of the corresponding MUC1-CD band to control for protein loading. The values were then graphed versus treatment and dose for each cell line. Click here for file

Additional file 2 **Growth curve of MUC1-CFP-Fv cells after treatment with AP20187^D ^or AP21998^M^**. Using Trypan blue exclusion assay, the number of live cells in a sample were counted daily for 3 days. The number of live cells in the sample was then extrapolated to estimate live cells in the population. No significant difference was found in the populations treated with AP21087^D^, AP21998^M^, or no treatment control. Click here for file
