# Supplementary figures and images for: Non-cysteine linked MUC1 cytoplasmic dimers are required for Src recruitment and ICAM-1 binding induced cell invasion
Source: Mol Cancer. 2011 Jul 28;10:93. doi: 10.1186/1476-4598-10-93 (PMC3161956; doi:10.1186/1476-4598-10-93)

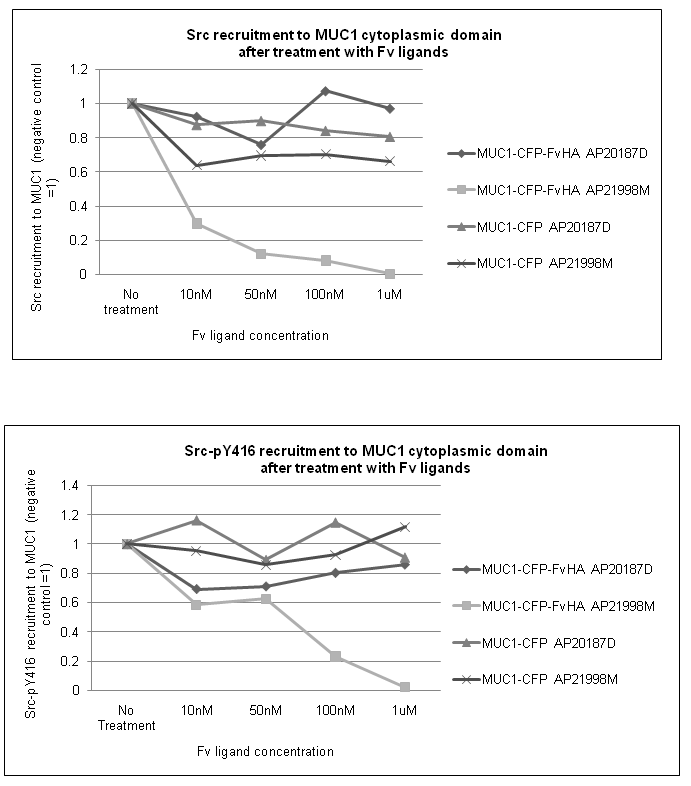

Supplement: Additional file 1 — Densitometry of Src and SrcP416 bands from SDS-PAGE (Figure 6) normalized to MUC1-CD. Using ImageJ software (NIH), the Src and Src P416 bands were analyzed for densitometric intensity, and values were normalized to the intensity of the corresponding MUC1-CD band to control for protein loading. The values were then graphed versus treatment and dose for each cell line. [file 1476-4598-10-93-S1.TIFF]

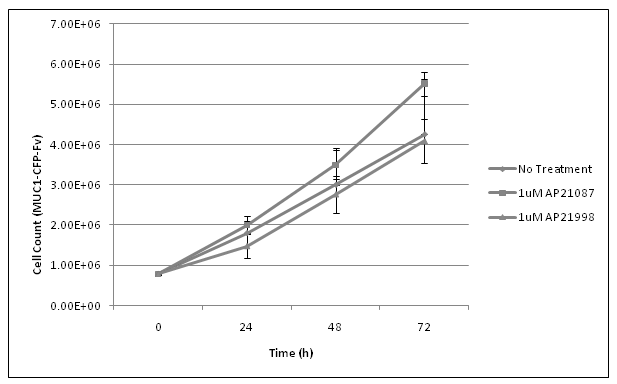

Supplement: Additional file 2 — Growth curve of MUC1-CFP-Fv cells after treatment with AP20187D or AP21998M. Using Trypan blue exclusion assay, the number of live cells in a sample were counted daily for 3 days. The number of live cells in the sample was then extrapolated to estimate live cells in the population. No significant difference was found in the populations treated with AP21087D, AP21998M, or no treatment control. [file 1476-4598-10-93-S2.TIFF]
